# Long noncoding RNA SNHG4: a novel target in human diseases

**DOI:** 10.1186/s12935-021-02292-1

**Published:** 2021-10-30

**Authors:** Qingfei Chu, Xinyu Gu, Qiuxian Zheng, Zixuan Guo, Dandan Shan, Jing Wang, Haihong Zhu

**Affiliations:** State Key Laboratory for Diagnosis and Treatment of Infectious Diseases, National Clinical Research Center for Infectious Diseases, Collaborative Innovation Center for Diagnosis and Treatment of Infectious Diseases, The First Affiliated Hospital, College of Medicine, Zhejiang University, NO. 79 Qingchun Road, Hangzhou, 310003 Zhejiang China

**Keywords:** LncRNA SNHG4, ceRNA, Human diseases, Function, Molecular mechanism

## Abstract

Recently, long noncoding RNAs (lncRNAs) have attracted great attention from researchers. LncRNAs are non-protein-coding RNAs of more than 200 nucleotides in length. Multiple studies have been published on the relationship between lncRNA expression and the progression of human diseases. LncRNA small nucleolar RNA host gene 4 (SNHG4), a member of the lncRNA SNHG family, is abnormally expressed in a variety of human diseases, including gastric cancer, renal cell carcinoma, glioblastoma, neuroblastoma, prostate cancer, colorectal cancer, osteosarcoma, cervical cancer, liver cancer, lung cancer, non-small-cell lung cancer, neonatal pneumonia, diabetic retinopathy, neuropathic pain, acute cerebral infarction, acute myeloid leukaemia, and endometriosis. In this paper, the structure of SNHG4 is first introduced, and then studies in humans, animal models and cells are summarized to highlight the expression and function of SNHG4 in the above diseases. In addition, the specific mechanism of SNHG4 as a competing endogenous RNA (ceRNA) is discussed. The findings indicate that SNHG4 can be used as a biomarker for disease prognosis evaluation and as a potential target for disease diagnosis and treatment.

## Introduction

RNA is mainly divided into coding RNA and noncoding RNA (ncRNA). According to transcriptome sequencing data, 70–90% of the human genome is involved in transcription; only 2% of the transcripts encode proteins, while the majority are non-protein-coding RNAs [1, 2]. ncRNAs principally include ribosomal RNA (rRNA), long noncoding RNA (lncRNA), transfer RNA (tRNA), microRNA (miRNA), small nuclear RNA (snRNA), circular RNA (circRNA), small nucleolar RNA (snoRNA), and piwi-interacting RNA (piRNA) [3]. Among them, lncRNAs, accounting for approximately 80% of ncRNAs [4], are the most studied. LncRNAs are ncRNAs that are more than 200 nucleotides in length and do not encode proteins [5, 6]. They have been reported to participate in many pathophysiological processes, including gene expression, protein activity, cell proliferation, apoptosis, and inflammation [7, 8]. Multiple studies have been published on the association between the expression of lncRNAs and the progression of human diseases.

LncRNA small nucleolar RNA host gene 4 (SNHG4) is a member of the SNHG family. SNHGs are the host genes of snoRNAs present in the nucleus and cytoplasm [9]. To date, it has been reported that the SNHG family has 22 members, from SNHG1 to SNHG22 [10]. They play significant roles in human cancers and other diseases. Xu et al. [11] pointed out that SNHG3 is a novel oncogenic lncRNA, which is aberrantly expressed in osteosarcoma, hepatocellular carcinoma (HCC), lung cancer, etc. Upregulation of SNHG3 contributes to biological functions, including tumour cell proliferation, migration, and invasion. Thin [12], Huang [13], and Xiao [14] have published reviews on SNHG1. They mainly summarized the relationship between SNHG1 and cancer and revealed that SNHG1 may act as a useful biomarker for the diagnosis, prognosis and treatment of human cancer. In addition, SNHG5, SNHG7, SNHG12, and SNHG16 have all been reported to promote the progression of cancers [15–18].

In this paper, we mainly summarized the research progress regarding SNHG4 in most tumours and some non-tumour diseases. Although an increasing number of studies on SNHG4 and human diseases have been published, they still do not cover all diseases. For example, there is no research report on SNHG4 in pancreatic cancer, breast cancer, etc. at present. In addition, there is a clear gap between existing research and clinical practice. In the future, more large-scale and multifaceted studies are needed to further verify the role of SNHG4 in various diseases. SNHG4 mainly plays a carcinogenic role in tumours. Reducing the expression of SNHG4 can inhibit the proliferation of tumour cells and is expected to become a potential target for cancer treatment. In some non-neoplastic diseases, in addition to affecting proliferation, SNHG4 is closely related to the immune response and can play dual pro-inflammatory and anti-inflammatory roles [20, 22]. In cerebral ischaemia–reperfusion injury, the SNHG4/miR-449c-5p/STAT6 axis participates in and inhibits the inflammatory process. In contrast, SNHG4 can also increase the levels of pro-inflammatory factors (IL-6, IL-12, and TNF-α) and promote neuroinflammation. In addition, Horikawa et al. [23] disclosed a transcript containing an intron sequence of SNHG4, which is expressed in podocytes. Podocytes play an indispensable role in the kidney [24]. Overall, we summarize the function of SNHG4 from many aspects, including human studies and in vivo and in vitro studies. Some mechanisms by which SNHG4 acts as a competing endogenous RNA (ceRNA) are also discussed, and it is finally speculated that SNHG4 may be used as a target for disease treatment, diagnosis and prognosis evaluation.

## LncRNA SNHG4, a member of the lncRNA SNHG family

**Fig. 1:**
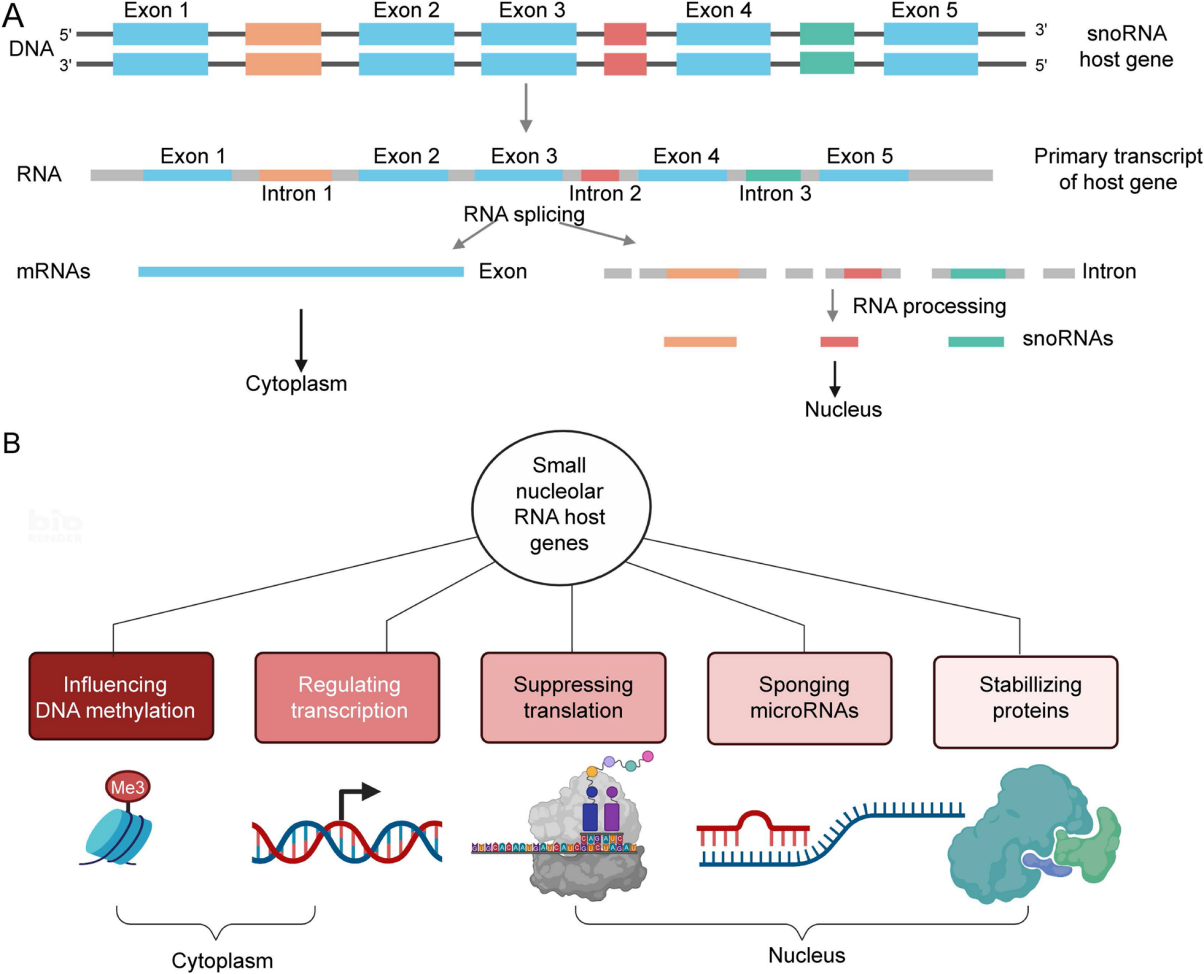
**A** The structure of the SNHG family members and the synthetic pathway of snoRNAs. **B** SNHGs are present in the cytoplasm and nucleus. They have five main types of molecular mechanisms of action

SNHGs are the host genes of snoRNAs (small nucleolar RNAs), including exons and introns [10, 25]. The introns are mainly processed into snoRNAs, while the exons are reassembled and play roles in the cytoplasm [10, 26]. Zimta et al. [27] first summarized the five main molecular mechanisms of SNHGs. SNHGs can influence DNA methylation, regulate transcription, repress translation, act as ceRNAs and prevent protein ubiquitination. LncRNA SNHG4, which is located at 5q31.2 [28], is a member of the SNHG family. SNHG4 also consists of exons and introns [23] (Fig. 1). In 2014, Chaudhry [29] first found that SNHG4 expression was increased in irradiated TK6 cells but was downregulated in bystander cells versus control cells. SNHG4 has been reported to be abnormally expressed in many diseases. It is downregulated in patients with neonatal pneumonia (NP) [30], diabetic retinopathy (DR) [19], acute cerebral infarction (ACI) [20], and acute myeloid leukaemia (AML) [21] and is upregulated in gastric cancer (GC) [31], renal cell carcinoma (RCC) [32], glioblastoma (GBM) [33], neuroblastoma [34], colorectal cancer (CRC) [35], etc. The aberrant expression of SNHG4 has been proven to be closely related to the genesis, occurrence and progression of various diseases.

## SNHG4 in human studies

### GC

GC, with its high incidence and mortality, has become the third leading cause of cancer-associated death worldwide [36, 37]. Due to the lack of specific symptoms in the early stage, most patients with GC are in the advanced stage at the time of diagnosis, and the treatment effect and prognosis are poor [38–40]. Thus, there is an urgent need to find new biomarkers for early diagnosis to achieve the best treatment response. Recently, lncRNAs have been constantly reported to play important roles during development and disease, including cancer [41–43]. Wang et al. [44] reported that SNHG4 was upregulated in GC tissues compared to normal adjacent tissue. The high expression level of SNHG4 was related to a lower survival rate. In addition, SNHG4 expression was noticeably associated with tumour-node-metastasis (TNM) stage (P < 0.001), lymph node metastasis (P = 0.003), and tumour invasion (P = 0.004). In summary, SNHG4 may act as an oncogenic lncRNA in GC.

### RCC

RCC, a renal-epithelial cancer, accounts for almost 90% of renal tumours [45, 46]. It is the second most common malignant cancer of the urinary system [47] and has a high distant metastasis rate. Currently, the most ideal treatment is standard nephrectomy; however, almost 25% of clear cell renal cell carcinoma (ccRCC) patients are prone to relapse after surgery [48, 49]. Therefore, it is significant to further explore the mechanisms of RCC and develop more effective target therapeutic drugs. The Cancer Genome Atlas (TCGA) data show that SNHG4 is upregulated in many cancers, including RCC [32], and Wu and colleagues [32] confirmed its high expression in RCC. Moreover, they further explored the correlation between SNHG4 and the clinical characteristics and prognosis of RCC patients. The results showed that high SNHG4 expression was closely related to relapse status, poor tumour grade, advanced T stage, lymph node invasion, and distant metastasis. Kaplan–Meier survival curve and multivariate Cox survival analysis both showed that high expression of SNHG4 predicted a poor prognosis. It was an independent prognostic factor for low survival rate and tumour relapse in RCC patients.

### GBM

Glioma is one of the most common types of primary intracranial carcinoma, and GBM is one of the gliomas [50]. GBM is highly malignant and aggressive, with a median survival of approximately one year [51, 52]. Despite therapies with surgery, radiotherapy, and chemotherapy, patient prognosis has not substantially improved [53]. It was reported that aberrant gene expression is vital in the development of GBM [54]. LncRNAs are a group of long-chain RNAs that do not encode proteins but can regulate gene expression. Lu [55] pointed out that LINC00511 facilitates temozolomide resistance in GBM cells by binding with miR-126-5p. In addition, studies have revealed that lncRNA HULC stimulates the epithelial-mesenchymal transition (EMT) process in human glioblastoma [56]. Wang and his team found [33] that SNHG4 is also upregulated in GBM. High expression of SNHG4 represents a worse prognosis.

### PCa

Prostate cancer (PCa) is one of the most universal malignant tumours [57, 58] and the fifth most common cause of cancer-related death in men [59]. Notably, approximately 15% of PCa cases are fatal, indicating that patients have a high risk of death. Despite the drastically improved PCa survival rate, patients at the metastatic stage still have a poor prognosis [60]. Thus, there is an urgent need to understand the molecular mechanism of PCa for the development of novel biomarkers that can be used for diagnosis and treatment [61]. Recently, it has been reported that lncRNAs can participate in the regulation of gene expression and changes in biological behaviour during the carcinogenesis of prostate cells [62]. Wang et al. [63] found that SNHG4 expression was higher in PCa tissues than in paracancerous tissues. High levels of SNHG4 decreased overall survival (OS) and were distinctly linked to lymph node metastasis (P = 0.029) and the tumour stage (P = 0.014) of PCa patients. SNHG4 is expected to become an effective biomarker for the diagnosis, treatment and prognosis of PCa.

### Osteosarcoma

Osteosarcoma is the most recognized primary malignant bone tumour and is mainly seen in adolescents [64, 65]; it is the second leading cause of cancer-related mortality among children and adolescents [66]. Due to the strong metastatic and highly invasive characteristics of osteosarcoma, the prognosis of patients is generally poor, and the five-year survival rate is only approximately 50% [67–69]. Thus, finding new molecular biomarkers for the diagnosis, treatment, and evaluation of metastasis is of great importance. Researchers used TCGA RNA-seq data to evaluate the expression of SNHG4 in human osteosarcoma samples. The results showed that SNHG4 expression was considerably increased in osteosarcoma tissues compared with adjacent non-tumour tissues. High SNHG4 expression was positively linked to tumour size (P = 0.020). Patients with high SNHG4 expression had a higher tumour recurrence and a lower survival rate [70]. Huang et al. [71] confirmed the above findings. In addition, they pointed out that a high level of SNHG4 is closely related to advanced pathological stage (P = 0.036) and distant metastasis (P = 0.039) in osteosarcoma patients.

### Liver cancer

Liver cancer is one of the most common cancers of the digestive system [72] and the second leading cause of cancer-related deaths worldwide [73–75]. In almost all patients, liver cancer is caused by chronic liver disease [76]. HCC is the most prominent histological form of liver cancer [76]. The economic and medical burden caused by liver cancer worldwide is very high [77]. ncRNAs are involved in the gene regulation of liver cancer. Researchers [78, 79] have discussed the role of SNHG4 in liver cancer. SNHG4 is upregulated in liver cancer. High SNHG4 expression was found to be correlated with histologic grade (P = 0.001), histological type (P = 0.01), stage (P = 0.01), survival status (P = 0.013), and T classification (P = 0.004). Compared with patients with low SNHG4 expression, those with high SNHG4 expression had poorer OS and relapse-free survival. Multivariate analysis further identified SNHG4 as an independent prognostic factor of poor survival in liver cancer.

### Other diseases

In ACI [20], NP [30], DR [19], AML [21], neuroblastoma [33], CRC [35], cervical cancer (CC) [80], non-small-cell lung cancer (NSCLC) [28], and endometriosis [81], researchers explored the expression of SNHG4 in patients with these diseases. However, they did not seem to have studied the relationship between SNHG4 and the clinical features of these diseases, nor have they explored the relationship between SNHG4 and disease prognosis. Overall, SNHG4 is abnormally expressed in a variety of diseases, suggesting that SNHG4 may be involved in their occurrence and development (Table 1). Further research on specific molecular mechanisms is expected to provide new ideas for the diagnosis and treatment of these diseases.

**Table 1:** The expression of SNHG4 and its clinical significance in a variety of diseases

Disease type	Number of clinical samples	Expression level	Clinicopathologic features	HR	P value	Prognostic implication of SNHG4 overexpression	Property	PMID
Gastric cancer (GC)	53 tissue samples from GC patients	Upregulation	Lymph node metastasis, tumour invasion, TNM stage	1.54	P < 0.001	Poor	Oncogene	33236157
Renal cell carcinoma (RCC)	99 pairs of RCC tissues and adjacent normal tissues	Upregulation	Advanced T stage, node invasion, distant metastasis status, poor tumour grade, relapse status	1.50	P = 0.007	Poor	Oncogene	33088220
Glioblastoma (GBM)	62 pairs of GBM tissues and adjacent normal tissues	Upregulation	–	3.32	–	Poor	Oncogene	32427712
Neuroblastoma	30 pairs of neuroblastoma tissues and adjacent normal tissues	Upregulation	–	–	P = 0.018	Poor	Oncogene	32614236
Prostate cancer (PCa)	113 pairs of PC tissues and adjacent normal tissues	Upregulation	Tumour stage, lymph node metastasis	–	P = 0.005	Poor	Oncogene	31608997
Colorectal cancer (CRC)	12 pairs of CRC tissues and adjacent normal tissues	Upregulation	–	–	–	–	Oncogene	33744866
Osteosarcoma	24 pairs of osteosarcoma tissues and adjacent normal tissues	Upregulation	Distant metastasis, lager tumour size, advanced pathological stage	–	P = 0.047	Poor	Oncogene	32537941
	136 cases of osteosarcoma patients and 40 adjacent normal tissue	Upregulation	Tumour size	–	P = 0.001	Poor	Oncogene	30152090
Cervical cancer (CC)	27 pairs of CC tissues and adjacent normal tissues	Upregulation	–	–	–	–	Oncogene	31590627
Liver cancer	371 liver cancer tissues and 50 normal liver tissues	Upregulation	Histological type, histologic grade, stage, T classification, survival status	–	P < 0.001	Poor	Oncogene	31967298
Hepatocellular carcinoma (HCC)	49 pairs of HCC tissues and adjacent normal tissues	Upregulation	–	–	P < 0.001	Poor	Oncogene	30537372
Non-small-cell lung cancer (NSCLC)	50 pairs of NSCLC tissues and adjacent normal tissues	Upregulation	–	–	–	Poor	Oncogene	33816782
Neonatal pneumonia (NP)	15 peripheral venous blood from NP patients and healthy controls	Downregulation	–	–	–	–	–	34450538
Diabetic retinopathy (DR)	60 tissue samples from DR patients, diabetic patients without complication and healthy controls	Downregulation	–	–	–	–	–	32454787
Acute cerebral infarction (ACI)	30 tissue samples from ACI patients and healthy controls	Downregulation	–	–	–	–	–	32982175
Acute myeloid leukaemia (AML)	60 tissue samples from AML patients and healthy controls	Downregulation	–	–	–	–	–	32319862
Endometriosis	25 EC samples and EU samples	Upregulation	–	–	–	–	–	32525017

EC, ectopic endometrium; EU, eutopic endometrium

## SNHG4 in in vivo studies

Some researchers have used xenotransplantation mouse models to explore the functional role of SNHG4 (Table 2). Cheng et al. [31] revealed that SNHG4 promotes GC cell growth in vivo. They injected HGC-27 and MKN-45 cells transfected with shNC and shSNHG4 subcutaneously into nude mice. Then, they measured the tumour size and weight of the two groups. Compared with the control, the tumours of the shSNHG4 group were significantly smaller and lighter. In addition, in an in vivo study of neuroblastoma, researchers found that SNHG4 promotes tumour cell growth, invasion, and migration, especially lung metastasis, and induces apoptosis [34]. Similarly, SNHG4 knockdown could inhibit RCC [32], CRC [35], osteosarcoma [71], CC [80], lung cancer [82], and NSCLC [28] tumour growth. In addition, Liu and colleagues [81] pointed out that silencing SNHG4 inhibited the growth of endometrial tissue outside the uterine cavity in vivo*.* In summary, the results of in vivo studies showed that SNHG4 not only plays a tumorigenic role in some cancers but also promotes the growth of intimal tissues in non-tumour diseases such as endometriosis. The development of drugs targeting SNHG4 has certain clinical value in the future.

**Table 2:** Function and mechanism of SNHG4 in different diseases

Disease type	Cell lines	Expression level	Effect in vitro	Effect in vivo	Genes/proteins/pathways affected	PMID
Gastric cancer (GC)	GES-1, AGS, HGC-27, NCI-N87, MKN-45, 293T	Upregulation	Proliferation ↑, migration ↑, invasion ↑, cell cycle arrest ↓	Tumour growth ↑	miR-204-5p, RRM2	33567852
	GES-1, SNU719, AGS, HGC-27	Upregulation	Proliferation ↑, migration ↑, invasion ↑, EMT ↑, apoptosis ↓	–	miR-204-5p	33236157
Renal cell carcinoma (RCC)	Caki-1, Caki-2, ACHN, 786-O, 769-P, HK-2	Upregulation	Proliferation ↑, migration ↑, invasion ↑, apoptosis ↓	Tumour growth ↑	miR-204-5p, RUNX2	33088220
Glioblastoma (GBM)	U-251	Upregulation	Proliferation ↑	–	miR-138, c-Met axis	32427712
Neuroblastoma	SH-SY5Y, CHP-212, SK-N-FI, and IMR-32, 293T	Upregulation	Proliferation ↑, migration ↑, invasion ↑, EMT ↑, apoptosis ↓	Tumour growth ↑ lung metastasis ↑	miR-377-3p	32614236
Prostate cancer (PCa)	PC-3, LNCaP, DU145, 22RV1, WPMY-1	Upregulation	Proliferation ↑, migration ↑, invasion ↑, apoptosis ↓	–	miR‐377, ZIC5	31608997
Colorectal cancer (CRC)	FHC, HCT8, LoVo, HCT116, SW620, HT29	Upregulation	Proliferation ↑, migration ↑, invasion ↑, cell cycle arrest ↓	Tumour growth ↑	miR-590-3p, CDK1	33744866
Osteosarcoma	hFOB1.19, HOS, MG63, Saos2, SJSA1, U2OS	Upregulation	Proliferation ↑, migration ↑, invasion ↑, apoptosis ↓	Tumour growth ↑	miR-377-3p	32537941
	MG-63, HOS, 143B, SW-1353, Saos2, U2OS	Upregulation	Proliferation ↑, colony formation ↑	–	miR-224- 3p	30152090
Cervical cancer (CC)	NCEC, c4-1, Caski, HeLa, SiHa	Upregulation	Proliferation ↑, apoptosis ↓	Tumour growth ↑	miR-148a-3p, c-Met	31590627
Lung cancer	NCI-H2170, NCI-H520, SK-MES-1, NCI-H1975, NCI-H1437, SPC-A-1	Upregulation	Proliferation ↑, migration ↑, invasion ↑, EMT ↑, cell cycle arrest ↓	Tumour growth ↑	miR-98-5p	31220419
Non-small-cell lung cancer (NSCLC)	H1299, H1650, H1975, SPCA1, 16HBE	Upregulation	Proliferation ↑, migration ↑, invasion ↑, apoptosis ↓	Tumour growth ↑	miR-let-7e, KDM3A, p21	33816782
Neonatal pneumonia (NP)	WI-38	Downregulation	Proliferation ↑, migration ↑, SOD concentration ↑, Inflammation ↓, apoptosis ↓	–	METTL3, NF-κB pathway	34450538
Diabetic retinopathy (DR)	ARPE-19	Downregulation	Apoptosis ↓	–	miR-200b, Oxr1	33822671
Neuropathic pain	PC12	Upregulation	Inflammation ↑	–	miR-423-5p	32454787
Acute cerebral infarction (ACI)	HAPI, HEK293	Downregulation	Inflammation ↓	–	miR-449c-5p, STAT6	32982175
Acute myeloid leukaemia (AML)	Kasumi-6	Downregulation	Proliferation ↓	–	miR-10a, PTEN	32319862
Endometriosis	HESCs	Upregulation	Proliferation ↑	Endometrial tissue growth ↑	miR-148a-3p, c-Met	32525017

## SNHG4 in cell lines studies

In addition to discussing SNHG4 expression and related clinicopathologic features for the diseases above, we summarized the potential biological functions and specific molecular mechanisms of SNHG4, which have been described in detail in in vivo studies. Next, we will mainly introduce the in vitro cell function verification of SNHG4. A large number of studies have revealed that SNHG4 mainly promotes proliferation, invasion, migration, apoptosis inhibition and EMT [31, 35, 70, 81]. In addition, Zhang and Pan et al. [20, 22] found that SNHG4 has dual pro-inflammatory and anti-inflammatory functions (Table 2).

### Proliferation

Cell proliferation is one of the most fundamental attributes of organisms [83]. Almost all living beings sustain life via cell proliferation [83]. This process also plays a vital role in the initiation and development of cancers [84, 85]. Researchers usually use Cell Counting Kit-8 (CCK-8), MTT, colony formation, and flow cytometry assays to assess the level of cell proliferation. Cyclin D1, Ki67, CDK1, CDK4, CDK6, and PCNA are all proliferation-related proteins, and their levels indirectly represent the level of cell proliferation. Cyclin D1 is a G1 checkpoint protein [86, 87]. Ki67, a well-known proliferation marker, can be used to evaluate cancer progression [88, 89]. CDK1, CDK4, and CDK6 are cyclin-dependent kinases that phosphorylate the corresponding proteins to drive the cell cycle process and thus affect cell proliferation [86, 90]. Proliferating cell nuclear antigen (PCNA) is also a cell proliferation-related marker [91, 92]. Cheng et al. [31] pointed out that, compared with the shNC group, the expression levels of cyclin D1, CDK6, and Ki67 were decreased in the shSNHG4 group. SNHG4 knockdown reduced cell viability and promoted cell cycle arrest, thus inhibiting GC cell proliferation. In CRC, researchers demonstrated an SNHG4/miR-590-3p/CDK1 axis that influenced the cell cycle and ultimately regulated cell proliferation [35]. SNHG4 binds to miR-590-3p to inactivate it, thereby alleviating the inhibitory effect of miR-590-3p on the downstream target gene CDK1. Finally, SNHG4 promotes the proliferation of CRC cells by promoting the transition of the cell cycle from the G2 phase to the M phase. In lung cancer, Tang and colleagues found that SNHG4 promotes cell growth by increasing the protein levels of Ki67, CDK4 and CDK6 [82]. SNHG4 was also found to promote the proliferation and migration of ectopic endometrium by regulating c-Met mediated by miR-148a-3p [81]. In addition, SNHG4 can function as a ceRNA, sponge miR-138 to upregulate c-Met [33], sponge miR-204-5p to upregulate RUNX2 [32], and then promote glioblastoma cell and RCC cell proliferation. Although SNHG4 plays a proliferation-promoting role in most diseases, it is downregulated in AML and inhibits proliferation by regulating the miR-10a/PTEN axis [21]. SNHG4 sponges miR-10a to upregulate PTEN, which acts as a tumour suppressor and can inhibit cancer cell proliferation.

In summary, the effect of SNHG4 on proliferation depends on the type of disease, although in most cases, it promotes proliferation. Future research can further explore the mechanisms and pathways by which SNHG4 affects proliferation.

### Migration, invasion and metastasis

Cell migration and invasion are two crucial initial processes of cancer cell metastasis [93, 94]. Cell migration is necessary for the normal growth and development of organisms. However, abnormal cell migration may lead to serious consequences such as tumour formation and metastasis [95]. Cell invasion refers to cell dissemination into adjacent organ tissues, thus inducing further development and distant metastasis of cancer cells [96]. Cell metastasis is the main cause of the widespread transformation of cancer cells to multiple organs [97]. All of these factors ultimately trigger cancer-related death. It has been reported that SNHG4 promotes migration, invasion, and metastasis in a variety of cancers. Cheng et al. [31] found that two metastasis markers, MMP2 and MMP9, were reduced after the knockdown of SNHG4, indicating that SNHG4 knockdown efficaciously decreases the metastatic capacity of GC cells. In addition, high expression of SNHG4 was associated with lung metastasis in neuroblastoma [34], lymph node metastasis in PCa [63], and distant metastasis in osteosarcoma [71].

EMT refers to the ability of epithelial cells to transform into mesenchymal cells during development [98, 99]. During this process, cancer cells lose cell-to-cell adhesion, gain the ability to migrate and invade, and finally trigger cancer cell metastasis [100, 101]. During EMT, E-cadherin expression levels are increased, while N-cadherin and Snail expression levels are decreased [102, 103]. In GC [44], neuroblastoma [34], and lung cancer [82], it has been reported that SNHG4 promotes EMT by upregulating E-cadherin and downregulating N-cadherin, thereby exerting a tumorigenic effect. Overall, SNHG4 plays an important role in invasion and migration.

### Apoptosis

Apoptosis, which is a type of programmed cell death (PCD), is a basic process of multicellular biological development [104, 105]. It can effectively remove damaged cells and maintain a stable internal environment. However, abnormal apoptosis can also trigger a series of diseases [106, 107]. Neurodegenerative diseases, anaemia, and transplant rejection are common causes of excessive apoptosis. Insufficient apoptosis can cause cancer and autoimmune diseases. Apoptosis is strictly controlled by multiple genes, including the Bcl-2 family, caspase family, some oncogenes, and the tumour suppressor gene P53 [108, 109]. As mentioned above, SNHG4 plays a carcinogenic role in most tumour diseases, and it can promote cancer cell proliferation and migration and inhibit apoptosis. SNHG4 silencing increases the expression of pro-apoptotic proteins such as Bax and caspase-3, thereby promoting neuroblastoma cell apoptosis [34]. Wu and colleagues also confirmed that overexpression of SNHG4 could inhibit RCC cell apoptosis [32]. Overexpression of SNHG4 resulted in an increase in Bcl-2 protein expression and a decrease in caspase-3, -8, and -9 activities in 769-P and ACHN cell lines, which proved that SNHG4 significantly inhibited the apoptosis of RCC cells. In another study on diabetic retinopathy, researchers found that SNHG4 upregulates the expression of Oxr1 by sponging miR-200b and thus inhibits ARPE-19 cell apoptosis [19]. In summary, SNHG4 acts to inhibit apoptosis in most diseases. Downregulating the SNHG4 level is expected to alleviate disease progression by promoting apoptosis.

### Inflammation

**Fig. 2:**
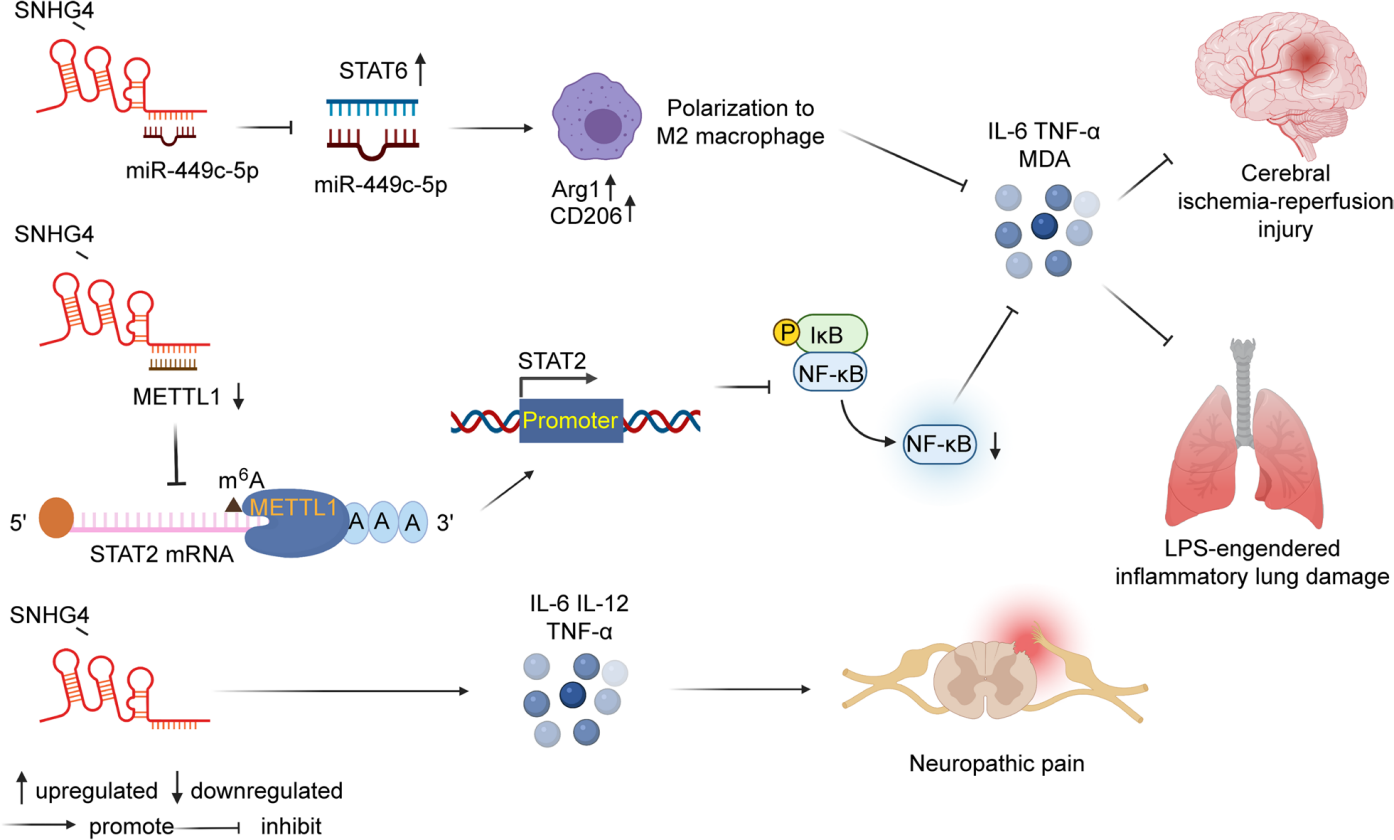
The dual role of SNHG4 on inflammation. In NP and ACI, SNHG4 had the effect of inhibiting inflammation. In neuropathic pain, SNHG4 promoted the expression of inflammation

Inflammation is the basic physiological response of the body to external stimuli; it is usually coordinated by immune cells to restore homeostasis [110]. In ACI, however, hyperactivated microglia produce massive amounts of inflammatory cytokines, causing inflammatory storms and eventually aggravating disease progression [111]. Zhang et al. [20] reported that SNHG4 can repress inflammation through the miR-449c-5p/STAT6 axis in microglia during cerebral ischaemia–reperfusion injury. The STAT signalling pathway is closely associated with immune regulation, and STAT6 is a cytoplasmic transcription factor that can be activated by IL-4 and IL-13. Their work revealed that the upregulation of SNHG4 inhibited the expression of miR-449c-5p and activated the STAT6 signalling pathway, thus inhibiting the M1 polarization of microglia and reducing inflammation. In NP, researchers found that SNHG4 inhibited the production of IL-6, TNF-α, and malondialdehyde (MDA), and inhibited the expression of NF-κB pathway proteins. Mechanistically, SNHG4 bound to and downregulated the expression of METLL3. METTL3 interference reduced the m6A level of STAT2 mRNA and promoted the STAT2 translation level. Finally, SNHG4 reduced the inflammatory lung injury caused by lipopolysaccharide (LPS) [30]. In another study on neuropathic pain, SNHG4 played the opposite role. During neuropathic pain progression, SNHG4 increases the levels of proinflammatory factors (IL-6, IL-12, and TNF-α) and promotes neuroinflammation [22]. In summary, SNHG4 can play a proinflammatory role and an anti-inflammatory role (Fig. 2), and the specific mechanism deserves further investigation.

## SNHG4 involved in ceRNA regulation

**Fig. 3:**
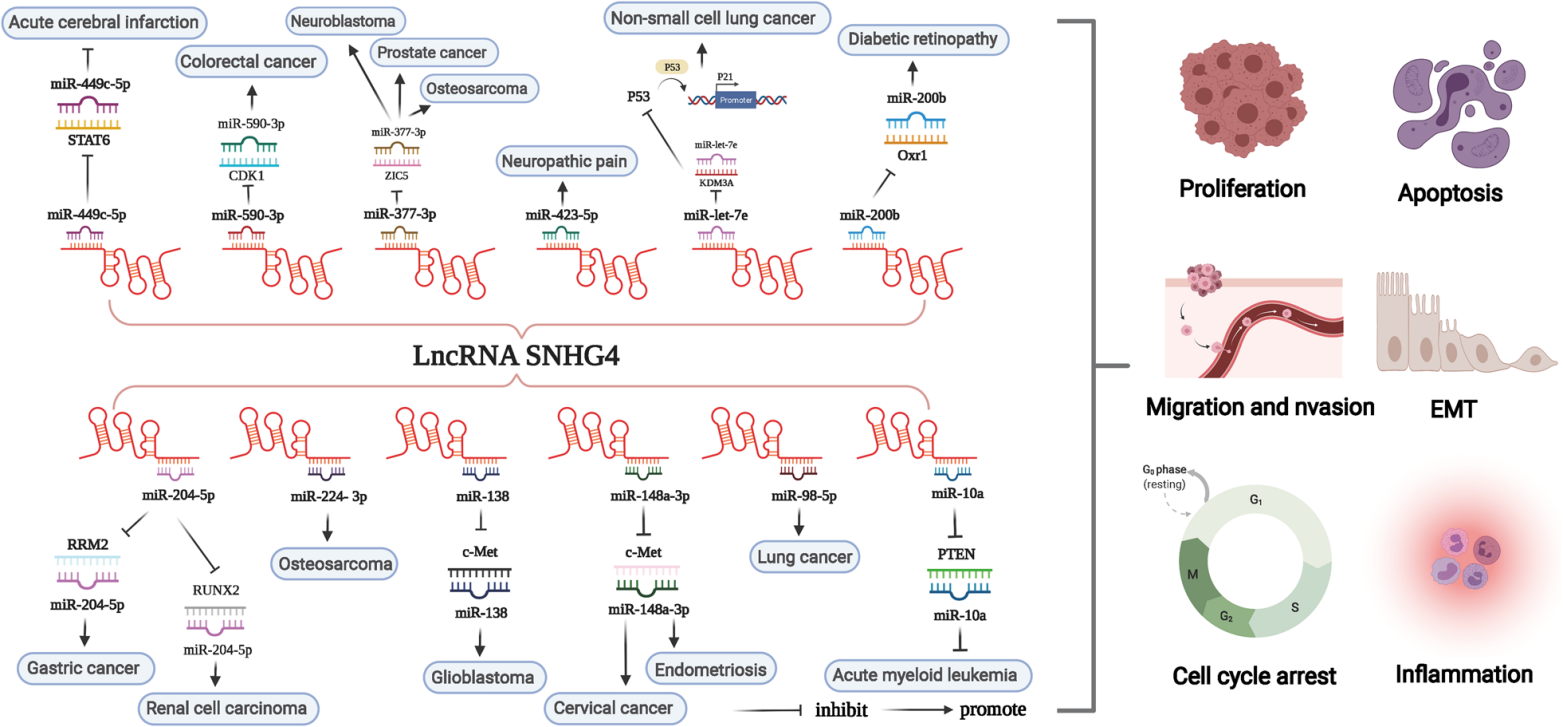
Function and molecular mechanisms of lncRNA SNHG4 in a variety of diseases. MiRNAs can cause gene silencing by binding to mRNAs, while ceRNAs can regulate gene expression by competitively binding to miRNAs. SNHG4 can serve as a ceRNA and sponge miRNA, thereby affecting the expression of target genes

In 2011, Salmena and colleagues first proposed a hypothesis about ceRNA: it forms a large-scale regulatory network that plays a role in both physiological and pathological conditions [112]. Theoretically, ceRNAs include all RNAs that contain microRNA response elements (MREs) and can recognize and bind miRNAs. At present, the most common ceRNAs are lncRNAs, circRNAs and pseudogene RNAs [113–115]. It is common knowledge that miRNAs mainly inhibit the expression of target genes by binding to mRNAs. ceRNAs can competitively bind to and inactivate miRNAs through MREs, thereby affecting the mRNA level of target genes [116–118]. Multiple studies have shown that lncRNAs, as ceRNAs, play an important role in malignant tumours and other diseases. This paper mainly summarized the lncRNA-miRNA-mRNA regulatory network formed by SNHG4 acting as a ceRNA (Table 2) (Fig. 3).

## Conclusions and perspectives

In recent years, there have been a variety of studies on lncRNAs. Researchers have focused on exploring the relationship between specific lncRNAs and diseases, especially cancer, as a breakthrough to provide new ideas for disease diagnosis and treatment [119]. Although lncRNAs do not encode proteins, they can regulate the expression of protein-coding genes in a variety of ways, most commonly through the ceRNA mechanism [120, 121]. LncRNAs can act as molecular sponges of miRNAs to negatively regulate their expression, resulting in the inhibition of miRNA targets, thus participating in a variety of important biological processes, such as embryonic development, stem cell maintenance, cell proliferation, differentiation, tumorigenesis, and cancer progression [122, 123].

SNHG4, a newly discovered lncRNA and one of the members of the SNHG family, has attracted great attention from researchers. First, researchers explored its expression in diseases and found that SNHG4 is highly expressed in most diseases and low in DR, ACI, and AML. They found that high expression of SNHG4 is closely associated with the clinicopathologic features and prognosis of some cancers. SNHG4 may therefore be used as a prognostic biomarker for these diseases. Next, they conducted a series of in vitro and in vivo studies to investigate the biological behaviour of SNHG4 in diseases. Experiments have proven that SNHG4 can promote cell proliferation, invasion, migration, and EMT and inhibit apoptosis. Targeted inhibition of SNHG4 seems to be beneficial for inhibiting the survival and development of tumour cells, thereby achieving therapeutic effects. Therefore, SNHG4 is expected to become a potential therapeutic target for various diseases. Finally, they revealed the molecular mechanism of SNHG4 in the pathogenesis of diseases. SNHG4 mainly acts as a sponge for miRNA. For example, SNHG4 sponges miR-204-5p and then upregulates RRM2 expression to exert a tumorigenic effect in GC. Similarly, the miR-148a-3p/c-Met axis and miR-let-7e/KDM3A/p21 axis play a role in the occurrence and development of CC and NSCLC, respectively.

Although there have been many types of research on SNHG4 and cancer, they are mainly in the basic research stage and more clinical application research needs to be carried out in the future. In addition, there are few reports of SNHG4 in non-neoplastic diseases; therefore, future research may be tilted towards non-neoplastic diseases. The development of lncRNA detection technology is very important for the diagnosis and treatment of diseases. The latest advances in CRISPR/Cas9 gene knockout, knock-in, and point mutation technologies may help us better understand the biological effects of lncRNAs to develop and apply clinically targeted therapeutic drugs. Currently, there is no approved or tested drug-related to SNHG4, however, as summarized in Fig. 3, SNHG4 regulates many miRNAs, thereby affecting the mRNA level of downstream target genes and promoting the expression of target genes. Targeted inhibition of SNHG4 or downstream genes such as STAT6, PTEN, RRM2, etc. may play an important role in inhibiting proliferation, invasion, migration, etc., and alleviate disease progression. In conclusion, SNHG4 is a novel target for disease diagnosis, treatment and prognosis evaluation, although the specific targeting mechanism and its true clinical application deserve further investigation.

## Data Availability

All data in our study are available upon request.

## Abbreviations

lncRNAs: Long non-coding RNAs
SNHG4: Small nucleolar RNA host gene 4
ceRNA: Competing endogenous RNA
ncRNA: Non-coding RNA
rRNA: Ribosomal RNA
tRNA: Transfer RNA
miRNA: MicroRNA
snRNA: Small nuclear RNA
circRNA: Circular RNA
snoRNA: Small nucleolar RNA
piRNA: Piwi-interacting RNA
NP: Neonatal pneumonia
DR: Diabetic retinopathy
ACI: Acute cerebral infarction
AML: Acute myeloid leukaemia
GC: Gastric cancer
RCC: Renal cell carcinoma
GBM: Glioblastoma
CRC: Colorectal cancer
TNM: Tumour-node-metastasis
ccRCC: Clear cell renal cell carcinoma
TCGA: The Cancer Genome Atlas
PCa: Prostate cancer
OS: Overall survival
HCC: Hepatocellular carcinoma
CC: Cervical cancer
NSCLC: Non-small-cell lung cancer
CCK-8: Cell counting kit-8
PCNA: Proliferating cell nuclear antigen
EMT: Epithelial-mesenchymal transition
PCD: Programmed cell death
MREs: MicroRNA response elements
MDA: Malondialdehyde
LPS: Lipopolysaccharide
